# Population structure analysis and genome-wide association study of a hexaploid oat landrace and cultivar collection

**DOI:** 10.3389/fpls.2023.1131751

**Published:** 2023-03-21

**Authors:** Lei Wang, Jinqing Xu, Handong Wang, Tongrui Chen, En You, Haiyan Bian, Wenjie Chen, Bo Zhang, Yuhu Shen

**Affiliations:** ^1^ Key Laboratory of Adaptation and Evolution of Plateau Biota, Northwest Institute of Plateau Biology, Chinese Academy of Sciences, Xining, China; ^2^ Qinghai Provincial Key Laboratory of Crop Molecular Breeding, Northwest Institute of Plateau Biology, Chinese Academy of Sciences, Xining, China; ^3^ Laboratory for Research and Utilization of Qinghai Tibetan Plateau Germplasm Resources, Northwest Institute of Plateau Biology, Chinese Academy of Sciences, Xining, China; ^4^ College of Life Sciences, University of Chinese Academy of Sciences, Beijing, China; ^5^ Innovation Academy for Seed Design, Chinese Academy of Sciences, Xining, China

**Keywords:** hexaploid oat, population structure, linkage disequilibrium (LD), genome-wide association analysis (GWAS), hullessness, lemma color

## Abstract

**Introduction:**

Oat (*Avena sativa* L.) is an important cereal crop grown worldwide for grain and forage, owing to its high adaptability to diverse environments. However, the genetic and genomics research of oat is lagging behind that of other staple cereal crops.

**Methods:**

In this study, a collection of 288 oat lines originating worldwide was evaluated using 2,213 single nucleotide polymorphism (SNP) markers obtained from an oat iSelect 6K-beadchip array to study its genetic diversity, population structure, and linkage disequilibrium (LD) as well as the genotype–phenotype association for hullessness and lemma color.

**Results:**

The average gene diversity and polymorphic information content (PIC) were 0.324 and 0.262, respectively. The first three principal components (PCs) accounted for 30.33% of the genetic variation, indicating that the population structure of this panel of oat lines was stronger than that reported in most previous studies. In addition, accessions could be classified into two subpopulations using a Bayesian clustering approach, and the clustering pattern of accessions was closely associated with their region of origin. Additionally, evaluation of LD decay using 2,143 mapped markers revealed that the intrachromosomal whole-genome LD decayed rapidly to a critical r^2^ value of 0.156 for marker pairs separated by a genetic distance of 1.41 cM. Genome-wide association study (GWAS) detected six significant associations with the hullessness trait. Four of these six markers were located on the Mrg21 linkage group between 194.0 and 205.7 cM, while the other two significant markers mapped to Mrg05 and Mrg09. Three significant SNPs, showing strong association with lemma color, were located on linkage groups Mrg17, Mrg18, and Mrg20.

**Discussion:**

Our results discerned relevant patterns of genetic diversity, population structure, and LD among members of a worldwide collection of oat landraces and cultivars proposed to be ‘typical’ of the Qinghai-Tibetan Plateau. These results have important implications for further studies on association mapping and practical breeding in high-altitude oat.

## 1 Introduction

Oat (*Avena sativa* L., 2n = 6x = 42) is an important cereal crop originating from the Mediterranean region (Montilla-Bascón et al., 2013), and an allohexaploid comprising three distinct subgenomes (A, C, and D) that arose through cycles of interspecific hybridization and polyploidization (Yan et al., 2016a). Oat is well-adapted to a cool climate (Hoffman, 1995), and is grown mostly in temperate regions of the world under a wide range of environmental conditions for food, feed, and forage. To date, oat has received considerable attention owing to its high nutritional value and the ability to reduce blood cholesterol levels and mediate the risk of cardiovascular disease (Anderson et al., 2009; Othman et al., 2011).

Compared with other staple cereal crops such as rice, maize, and wheat, the breeding, genomics, and population structure analyses of oat have been lagging, primarily owing to its large, repeat-rich, and polyploid genome and low investment (Tinker et al., 2009; Yan et al., 2016b). Advances in molecular marker exploitation technology have enhanced genome-wide marker discovery in oat. Valuable studies have been carried out on oat genetic diversity, population structure, quantitative trait locus (QTL) identification, and genotype–phenotype association using various molecular markers, including amplified length fragment polymorphism (ALFP) (Achleitner et al., 2008), random amplified polymorphic DNA (RAPD) (Ruwali et al., 2013), simple sequence repeat (SSR) (Montilla-Bascón et al., 2013), diversity arrays technology (DArT) (Tinker et al., 2009; He and Bjørnstad, 2012), and single nucleotide polymorphisms (SNPs) (Winkler et al., 2016; Cömertpay et al., 2018; Yan et al., 2020). A dense consensus map of oat with 12,000 markers based on 12 biparental populations was recently constructed (Chaffin et al., 2016) and supplemented by high-density SNPs discovered through genotyping-by-sequencing (GBS) (Huang et al., 2014; Bekele et al., 2020). The availability of dense markers opens new opportunities for association mapping, molecular breeding, genetic diversity analysis, genome sequencing, and map-based cloning in oat (Chaffin et al., 2016). Moreover, great progress has been recently made in oat genome sequencing and assembly. Four chromosome-scale genome assemblies of diploid, tetraploid, and hexaploid oat have recently been reported (Maughan et al., 2019; Li et al., 2021; Peng et al., 2022; https://wheat.pw.usda.gov/GG3/graingenes_downloads/oat-ot3098-pepsico). These reference genomes will accelerate the studies on oat evolution and gene identification.

Studies show that population structure in oat is not as strong as that in other crops (Winkler et al., 2016; Peng et al., 2022). No one factor, such as geographical origin or morphological traits (such as hulled or hulless grains, lemma color, and panicle type), significantly affect population stratification patterns (Montilla-Bascón et al., 2013; Esvelt Klos et al., 2016).

With the advent of rapid genotyping and next-generation sequencing technologies, genome-wide association study (GWAS) has emerged as a powerful routine strategy to identify genes or regions affecting complex traits in crop species (e.g., Huang et al., 2010, for rice agronomic traits; Wang et al., 2012, for resistance to head smut in maize; Alqudah et al., 2014, for photoperiod response in barley) over the last decade. In oat, GWAS has been performed to study agronomic traits (Winkler et al., 2016; Tumino et al., 2017), quality traits (Newell et al., 2012; Asoro et al., 2013; Carlson et al., 2019), and biotic or abiotic stress tolerance (Tumino et al., 2016). Six significant associations for lodging and two for plant height were detected by Tumino et al. (2017) in a European oat collection using the 6K SNP array. Three independent markers were significantly associated with β-glucan concentration, and one showed sequence homology to genes in rice (Newell et al., 2012). All of these studies indicated that GWAS was an effective method for QTL detection in oat.

The objectives of the present study were to (1) assess the genetic diversity of an oat collection originating from globally diverse regions; (2) characterize the population structure of the oat germplasm; (3) evaluate the extent of pairwise linkage disequilibrium (LD); and (4) perform GWAS for studying morphological traits. The results of this study would be useful for a deeper understanding and better management of the different kinds of oat genetic resources. This study provides valuable genetic markers for oat breeding programs, and represents a successful example for further association studies in oat.

## 2 Materials and methods

### 2.1 Plant material

A collection of 288 oat landraces and cultivars was used in this study. Out of 288 oat accessions, 257 accessions (199 landraces and 58 cultivars), originating from 34 countries, were obtained from the USDA National Small Grain Collection (NSGC) (**Figure S1** and **Table S1**). In addition, 29 commercial oat cultivars and two mutagenized genotypes were collected from the oat-producing provinces of northern China (Inner Mongolia, Hebei, Qinghai, Gansu, and Jilin). Further details of the improvement status, country of origin, growth habit, hull type (hulled or hulless), and lemma color of these accessions are provided in **Table S1**. The ‘country of origin’ information was used to assign each accession to a region of origin as defined by the United Nations Statistics Division.

### 2.2 Genotypes

Genomic DNA of each accession was extracted from bulked leaf samples of 2-week-old seedlings using the Plant Genomic DNA Kit (Qiagen Inc., USA). The concentration and quality of each DNA sample were assessed by agarose gel electrophoresis and with a nanophotometer (NanoDrop 2000C, Thermo Scientific, USA). A total of 4,852 SNP markers were assayed using the oat iSelect 6K-beadchip array (Illumina, San Diego, CA, USA) at the USDA-ARS Genotyping Laboratory at Fargo, ND, USA, as described by Tinker et al. (2014). SNP genotype calls were made and adjusted in GenomeStudio v2011.1 (Illumina, San Diego, CA, USA). The SNP filtering process was performed according to the requirements of the bioinformatics analysis. The following were eliminated: multiallelic and monomorphic SNPs; SNPs with poor genotype calls resulting from weak signal or ambiguous clustering; and SNPs with relative minor allele frequency (MAF) ≤ 0.05 and missing data > 0.1. The position information of SNPs used in the present study was obtained from the consensus map of oat (version 3.1; Chaffin et al., 2016). The consensus map contains 21 linkage groups, scaled by genetic distance (cM). Linkage groups that are the consensus of the underlying component maps are designated by Merge (Mrg) and are reffered as Mrg01 to Mrg33.

### 2.3 Morphological trait data and phenotypic analysis

Morphological trait data, including hullessness and lemma color, were downloaded from the Germplasm Resources Information Network (GRIN; https://npgsweb.ars-grin.gov/gringlobal/search) on November 20, 2020, and the traits were affirmed by field planting in the summer of 2020. Oat accessions were planted in April at Diyao Village, Huangzhong County, Qinghai Province (N 36°29′03.63″, E 101°31′09.91″). Each accession was sowed two rows at a sowing density of 20 grains per row. Rows were seperated from each other by 20 cm. At maturity period, the hullessness trait of oat accessions was recorded. If the caryopsis of an oat accession is tightly surrounded by thick, lignin-rich hull after handy threshing, the accession is reffered to as hulled oat and recorded as “Hulled”; whereas if the hull of oat accession is papery and free-threshing, the accession is reffered to as hulless oat and recorded as “Hulless”. At milk-ripe stage, the lemma color of oat accessions is observed and the color is recorded as “Amber/White”, “Black”, “Grey”, “Red”, “Yellow”.

### 2.4 Genetic diversity, population structure, and LD analyses

Statistics including genetic diversity and polymorphic information content (PIC) were calculated for each locus using the PowerMarker v3.25 software (Liu and Muse, 2005). To estimate the population structure, three methodologies were compared. Model-based structure analysis was performed using STRUCTURE (Pritchard et al., 2000) with the number of ancestral populations (K) ranging from 2 to 10, and the number of subgroups was identified. Principal component analysis (PCA) was carried out using the GCTA software (Yang et al., 2011), and the percentage of genotypic variation explained by the first three PCs is shown in section 3.2 to enable comparison with the data obtained in previous oat studies. In addition, a neighbor-joining tree was constructed using MEGA6 (Tamura et al., 2013) with 1,000 bootstrap replicates.

Pairwise LD was estimated using squared allele frequency correlation (r^2^) based on loci that have been mapped on the consensus map. The r^2^ values were calculated using the LDcorSV package of R (Mangin et al., 2012). The genome-wide and chromosomal LD decay data were plotted against the genetic distance (cM), and the LOESS curve was fitted using R.

### 2.5 GWAS

To evaluate genotype–phenotype associations, GWAS was performed using a mixed linear model (MLM) implemented in TASSEL v5 (Bradbury et al., 2007), with default settings. The PCA matrix and kinship information (K matrix), generated using GCTA and TASSEL v5, respectively, were incorporated in the MLM as covariates. Quantile-quantile (Q-Q) plots and Manhattan plots were generated using the qqman package of R. According with the SNP annotations provided by Tinker et al. (2014), genes orthologous to those carrying the trait-associated SNPs are detected. The design sequence of significant associated SNP are aligned to the new reference genome assembly of hulless common oat (Peng et al., 2022) and the position of trait-associated SNPs in the common oat physical map are determined. The physical chromosomes are nominated as A1-A7, B1-B7, D1- D7.

## 3 Results

### 3.1 Genetic diversity

A total of 3,313 polymorphic SNPs were obtained using the 6K Illumina platform. After filtering, 2,213 SNPs with no more than 10% missing calls and at least 5% MAF were retained, of which 2,143 SNPs were mapped on to the consensus map across all 21 linkage groups, covering a total genetic distance of 2688.3 cM with an average spacing of 1.25 cM between two SNPs. The number of SNPs within and among linkage groups varied from 42 (on linkage group Mrg19) to 211 (Mrg01) (**Table 1**). These markers showed an average genetic diversity of 0.324, with a mode ranging from 0.096 to 0.50. The PIC value varied from 0.091 to 0.375 (average PIC = 0.262). Mean genetic diversity and PIC values, calculated for each of the 21 chromosomes, were found to be similar within the chromosomes (**Table 1**). The average PIC of oat landraces was 0.260, and that of oat cultivars was 0.259. There was no significant difference between landrace and cultivar of oat.

**Table 1:** SNP marker distribution and coverage, gene diversity, and polymorphic information content (PIC) across all linkage groups in 288 oat accessions.

Linkage group	No. of SNP markers	Chromosome length (cM)	Marker coverage	Gene diversity	PIC
Mrg1	211	143.3	0.68	0.3040	0.2484
Mrg2	143	111.7	0.78	0.3048	0.2497
Mrg3	110	167.8	1.53	0.3391	0.2727
Mrg4	78	61.1	0.78	0.3517	0.2829
Mrg5	88	154.5	1.76	0.3556	0.2828
Mrg6	98	146.8	1.50	0.3236	0.2625
Mrg8	136	186.4	1.37	0.2955	0.2443
Mrg9	95	129	1.36	0.3383	0.2730
Mrg11	144	108.6	0.75	0.3212	0.2616
Mrg12	92	107.9	1.17	0.3127	0.2554
Mrg13	89	119.2	1.34	0.3418	0.2753
Mrg15	77	88.2	1.15	0.3221	0.2606
Mrg17	119	114.5	0.96	0.3422	0.2755
Mrg18	64	90	1.41	0.3223	0.2618
Mrg19	42	78.5	1.87	0.2983	0.2450
Mrg20	151	251.1	1.66	0.3430	0.2758
Mrg21	116	212.1	1.83	0.3130	0.2545
Mrg23	52	101.9	1.96	0.3366	0.2692
Mrg24	121	95.3	0.79	0.3408	0.2743
Mrg28	63	95.6	1.52	0.3242	0.2628
Mrg33	54	124.8	2.31	0.3378	0.2724
Total	2143	2688.3	1.25	0.3271	0.2648

### 3.2 Population structure

**Figure 1:**
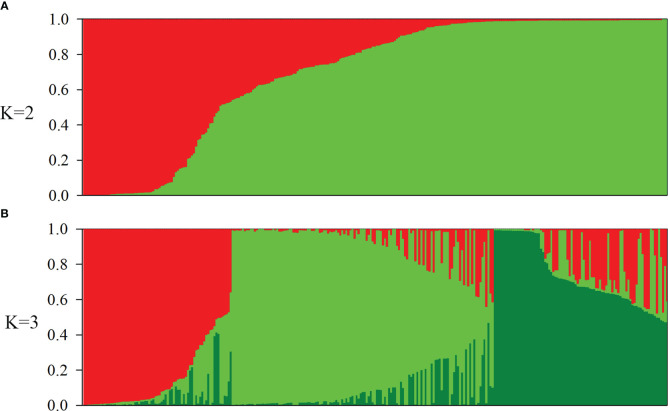
Population structure of 288 oat (*Avena sativa*. L) accessions. **(A, B)** Population structure estimated using STRUCTURE and illustrated as bar plots (K = 2 and 3). Each accession is shown as a thin vertical segment, and the color indicates the proportion of each population. Each subgroup is represented by an individual color.

Population structure in the oat germplasm was investigated using the model-based method implemented in the STRUCTURE software, which assigns each individual a membership coefficient for each cluster. Following the method of Evanno et al. (2005), the optimal number of populations (K) was estimated using the results exported from STRUCTURE. The maximum delta K (ΔK) value was inferred to be two, suggesting that K=2 was the most likely value for the oat collection, with K = 3 being the second best(**Figure S2**). Accessions with the probability of membership to either population greater than 0.7 were assigned to that specific population, and those with membership probability less than 0.7 were considered admixtures. According to these criteria, 239 of 288 accessions (82.99%) were assigned to one of the two populations (POP1 and POP2), while the remaining 49 accessions (17.01%) were retained in the admixed group (Admixed) (**Figure 1A**, **Table 2**). Among the 288 accessions, 56 hulled accessions (48 landraces and 8 cultivars) were assigned to POP1. The landraces in POP1 mainly originated from Western Asia (27), Southern Europe (10), and Southern Asia (6). POP2 consisted of 130 hulled landraces, 45 hulled cultivars, and 8 hulless cultivars. The landraces in POP2 were mainly from Eastern Asia (24), Eastern Europe (49), Southern Europe, (32) and Southern America (11). The cultivars in POP2 were principally from Eastern Asia (29), Northern America (10), and Eastern Europe (8). Among the 31 cultivars collected from North China, 20 including 8 hulless lines were classified into POP2, while the remaining 9 cultivars were considered admixed. Using K = 3, the population POP2 was further divided into two subpopulations; however, the majority of POP2 accessions, especially cultivars, fell into the admixed group (**Figure 1B**, **Table 2**).

**Table 2:** Grouping of 288 oat accessions based on STRUCTURE analysis at K = 2 and K = 3.

Region of origin	POP1^a^		POP2^b^		Total	POP1		POP2		POP3		Total
	Land.	Cult.	Land.	Cult.		Land.	Cult.	Land.	Cult.	Land.	Cult.	
Eastern Asia			24	29	53			19	2	3	9	33
Western Asia	27		11		38	26		11				37
Southern Asia	6		2		8	6		2		1		9
Central Asia			1		1			1				1
Eastern Europe	1	2	49	8	60	1	2	34	3	12	3	55
Western Europe		1		1	2		1		1			2
Southern Europe	10		32		42	10		32		1		43
Northern Europe				3	3				2		1	3
South America	3		11		14	3		2		6		11
Northern America		3		10	13		3		4		3	10
Eastern Africa	1				1	1						1
Northern Africa				1	1							0
Oceania		2		1	3		2				1	3
Total	48	8	130	53	239	47	8	101	12	23	17	208

a POP1, population 1; Land., number of landraces; Cult., number of cultivars.

b POP2, population 2.

**Figure 2:**
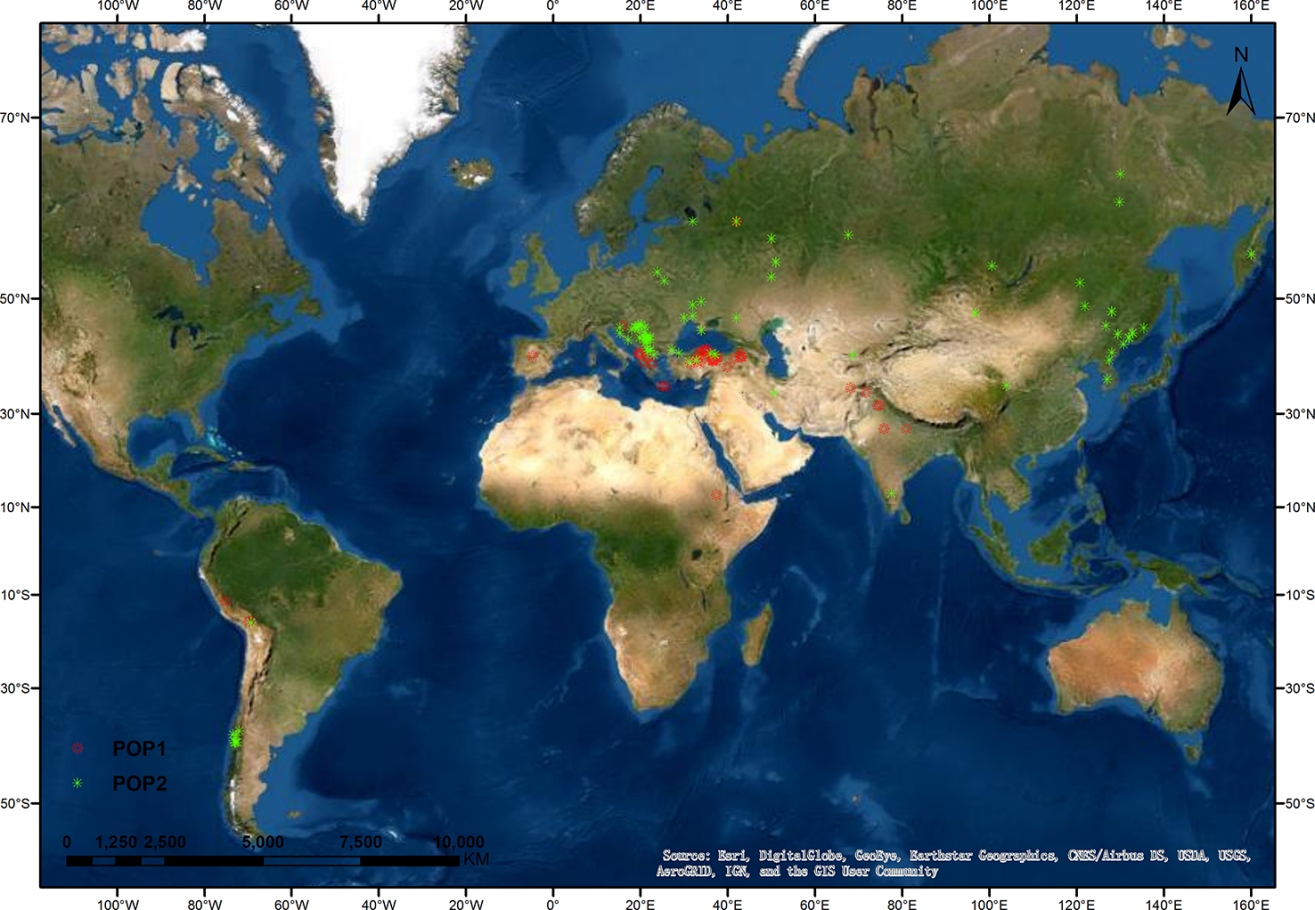
Geographical distribution of oat landraces at K = 2. The locations of oat landraces, with population membership coefficient ≥ 0.7, are indicated by different colors.

Next, we analyzed the correlation of the membership coefficients of accessions with their region of origin, improvement status, year of receipt, and lemma color. The correlation coefficient was highest between membership coefficients and origin regions (0.50, *P* < 0.001), and this correlation in landraces was up to 0.55 (*P* < 0.001) (**Table S2**). We also analyzed the distribution of landraces in the two populations (**Figure 2**, **Table 2**). All landraces from Eastern Asia, the overwhelming majority of landraces from Eastern Europe (49 of 50), and a large part of landraces from Southern Europe (32 of 42) and Southern America (11 of 14) were assigned to POP2. Landraces from Western Asia were divided into the two populations (27, POP1; 11, POP2). Most of the landraces from Southern Asia (6 of 8) and a minority of accessions from Southern Europe (10 of 42) were assigned to POP1. Overall, the landrace accessions in POP1 were distributed in lower latitude regions compared with those in POP2 (**Figure 2**).

**Figure 3:**
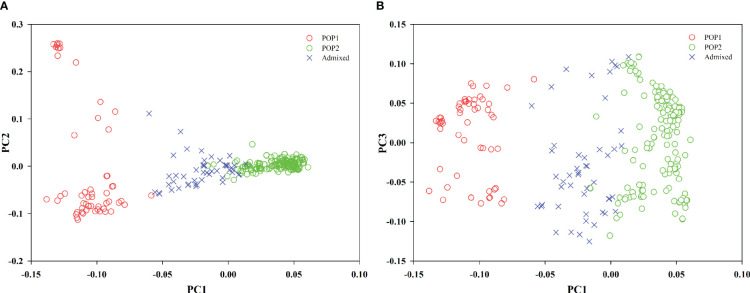
Scatterplots showing the results of principal component analysis (PCA) conducted based on the SNP marker data of oat lines labeled according the different subgroups identified by STRUCTURE (membership coefficient ≥ 0.7). **(A, B)** Population stratification of oat lines according to the first component PC1 vs. PC2 **(A)**, and PC1 vs. PC3 **(B)**. Admixed individuals are indicated with ×.

PCA was also used to infer the population structure of the oat germplasm. The first three principal components (PC1–PC3) together accounted for 30.33% of the genetic marker variation (PC1, 16.26%; PC2, 8.05%; PC3, 6.02%). Two two-dimensional (2-D) scatter plots of the 288 oat genotypes (**Figure 3**) exhibited a similar population stratification to that of STRUCTURE. PC1 clearly separated POP1 from POP2, and some accessions, identified as admixed in STRUCTURE, were placed in intermediate positions in the PCA plot (**Figure 3**).

**Figure 4:**
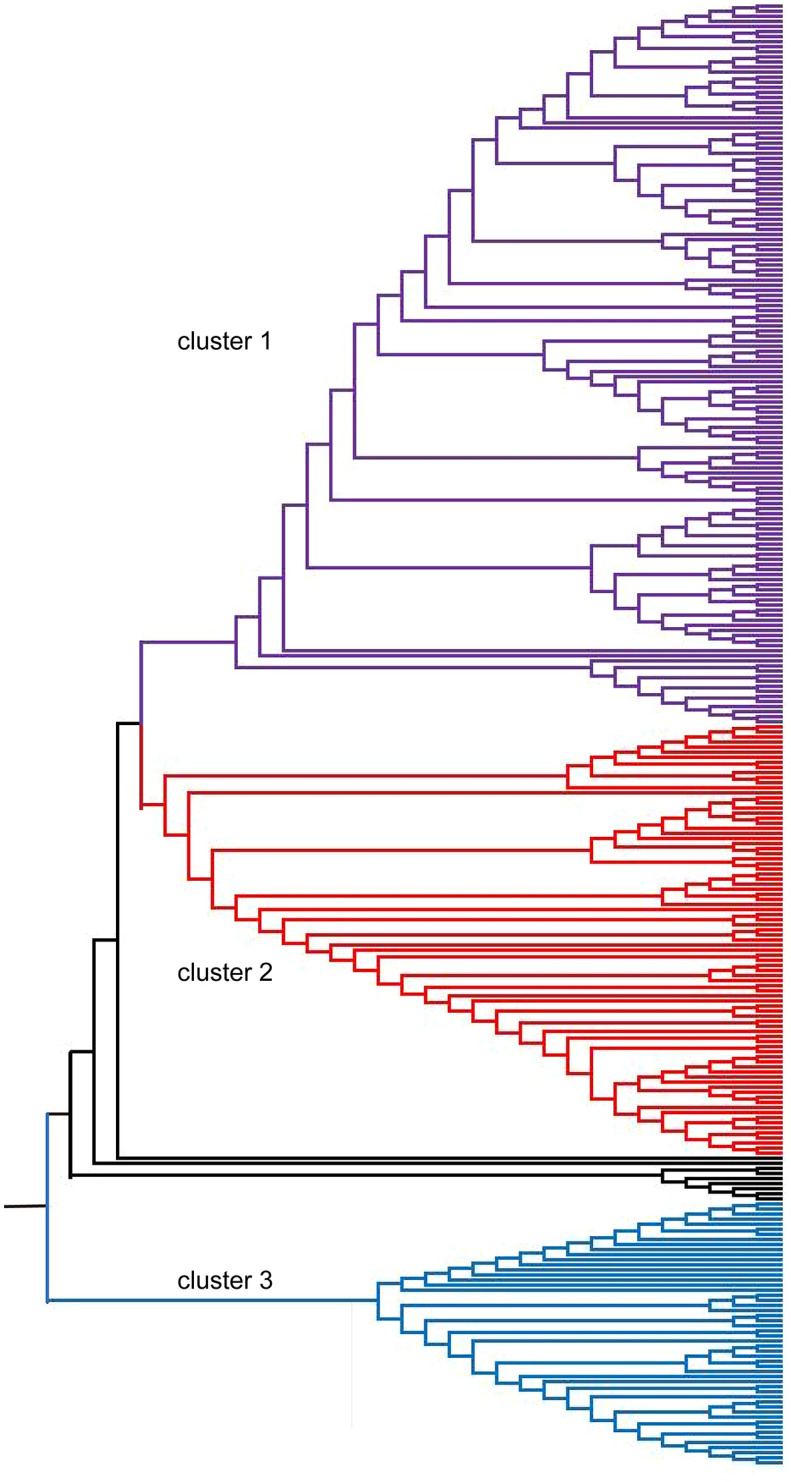
Neighbor-joining phylogenetic tree of 288 oat accessions based on 2,213 SNPs. Each accession is denoted as a vertical line in three colored subclades corresponding to the three clusters.

The stratification of the oat germplasm was further determined by the neighbor-joining (NJ) method implemented in the program MEGA 6.0 (**Figure 4**), and the accessions were divided into three clusters. Consistent with the results of STRUCTURE, most oat accessions from Eastern Asia, Eastern Europe, and Southern America grouped into Cluster 2 (**Supplementary Table S3**). Unlike the STRUCTURE results, most oat lines from Western Asia grouped into Cluster 1 (**Table S3**). Generally, the stratification of accessions was associated with their geographical origin.

The PIC of POP1 (0.2621) was greater than that of POP2 (0.2348), indicating that POP1 was genetically more diverse than POP2. Highly significant (*P* < 0.001) genetic variance resided among the two populations (17.34%). The estimated fixation index (*F*st = 0.1379) was also highly significant (*P* < 0.001). According to the degree of population genetic variance corresponding to *F*st values (Hartl et al., 1997), there was moderate genetic variance between the two populations.

### 3.3 LD analysis

The 2,143 mapped SNPs were used to explore the LD level in the present oat panel. The r^2^ values revealed a high degree of association among many unlinked and loosely linked markers within all chromosomes (**Figure S3**). A critical value of r^2^ beyond which LD is likely to be caused by genetic linkage was calculated by resampling unlinked markers, and was fixed at 0.156 (Breseghello and Sorrells, 2006). The r^2^ values for intrachromosomal locus pairs ranged from 0 to 1, with an average of 0.085. Of these r^2^ values, 15.36% exceeded 0.156 and averaged to 0.365. The genome-wide and intrachromosomal LD decayed rapidly with genetic distance (**Figure S4**). The point at which the LOESS curve and the line r^2^ = 0.156 intersected was considered the average LD decay distance. Based on the criteria, the average genome-wide LD decay distance was 1.41 cM, and the intrachromosomal LD decayed between 0.02 and 14.99 cM (**Figure S4**, **Table S4**). The different chromosomes showed different LD levels, indicating that they had been subjected to variable intensities of natural and artificial systematic selection.

### 3.4 Genome-wide association

GWAS was performed using 2,143 mapped SNPs. This number of markers was used to establish the threshold of statistical significance of association at *p* ≤ 2.33 × 10^-5^, calculated by applying the Bonferroni correction with an experiment-wise α = 0.05. At *p* < 2.33 × 10^-5^, nine SNP markers showed significant association with the grain hull type and lemma color of oat accessions, and explained 8.28–22.39% of the phenotypic variation in these traits (**Table 3**; **Table S5**).

**Table 3:** Hullessness- and lemma color-associated SNPs and their positions in the oat consensus genetic map.

Trait	SNP marker	Linkage group	Position (cM)	*P*-value	R^2^ (%)
**Hullessness**	GMI_GBS_84661	Mrg21	194	1.02E-11	22.39
	GMI_ES01_c8241_504	Mrg21	194	2.13E-11	17.11
	GMI_GBS_67251	Mrg21	205.7	8.02E-07	8.95
	GMI_ES22_c7478_431	Mrg21	205.7	7.57E-06	8.36
	GMI_ES02_lrc13788_346	Mrg5	131.6	6.10E-06	8.80
	GMI_ES17_c10594_472	Mrg9	61.8	1.26E-05	8.28
**Lemma Color**	GMI_ES15_c2369_181	Mrg20	14.7	4.86E-11	21.16
	GMI_DS_oPt-18257_376	Mrg17	53.8	5.58E-08	12.68
	GMI_GBS_13773	Mrg18	56	1.42E-06	9.86

**Figure 5:**
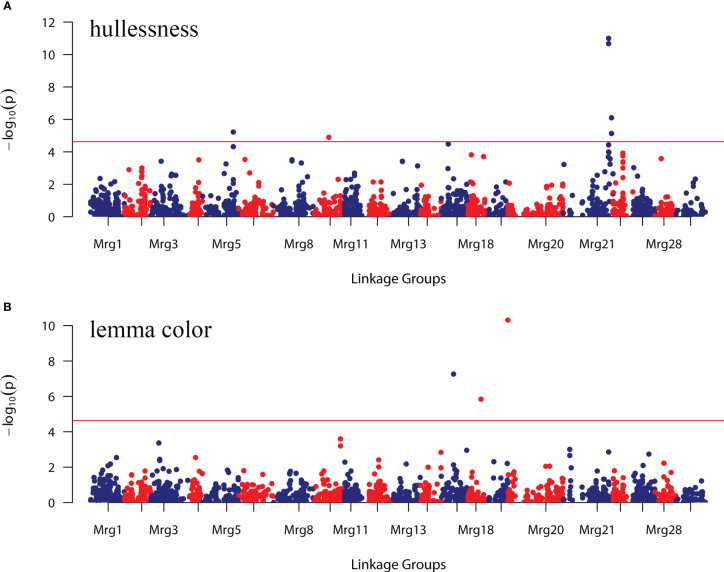
Genome-wide association study (GWAS) of the hullessness and lemma color traits in the oat collection using 2,143 SNP markers. **(A)** Hullessness; **(B)** lemma color. The red line represents the threshold calculated according to the false discovery rate (FDR). Markers above the red line in **(A, B)** were significantly associated with the respective trait.

**Figure 6:**
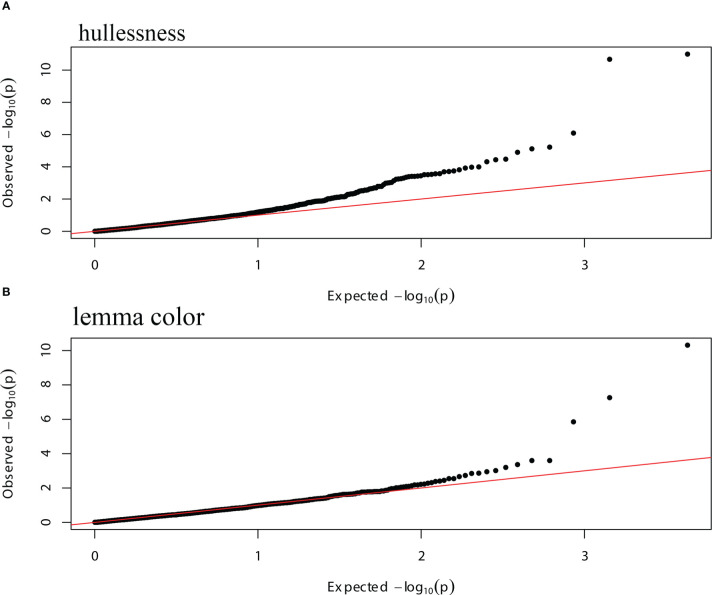
Quantile-quantile (Q-Q) plots of the GWAS data of hullessness and lemma color using a mixed model, with the PCA matrix and kinship information as covariates. **(A)** Hullessness; **(B)** lemma color.

The strongest evidence of association with the hullessness trait was observed on linkage group Mrg21, specifically based on two markers, GMI_GBS_84661 and GMI_ES01_c8241_504, both located at 194 cM, with the minimum *p*-value less than 1.0 × 10^-10^ (**Figures 5A**, **6A**). Strong associations for this trait were also found on linkage group Mrg21 (GMI_GBS_67251 and GMI_ES22_c7478_431, comapping at 205.7 cM, *p* < 1.0 × 10^-5^). Two additional markers, one mapped on Mrg05 (GMI_ES02_lrc13788_346, *p* < 1.0 × 10^-5^) and the other on Mrg09 (GMI_ES17_c10594_472, *p* < 1.0 × 10^-6^), were also significantly associated with hullessness (**Figure 5A**, **6A**).

Three significant markers were identified for lemma color. Among these, GMI_ES15_c2369_181 (Mrg20, 14.7 cM) showed the most significant effect on lemma color (*p* < 1.0 × 10^-10^) (**Figures 5B**, **6B**). The other two markers significantly associated with lemma color were GMI_DS_oPt-18257_376 (Mrg17, 53.8 cM) and GMI_GBS_13773 (Mrg18, 56 cM) (**Figures 5B**, **6B**).

## 4 Discussion

### 4.1 Patterns of genetic diversity and population structure

Oat has not only been grown as a grain or forage crop but it has also received significant attention as a whole-grain food owing to its health benefits for humans (Newell et al., 2011). Approximately 80,000 oat cultivars and over 20,000 wild oat accessions have been preserved in gene banks, and are considered as a pool of potentially useful genes (Lipman et al., 2005). Genetic diversity and population structure studies of the oat germplasm provide important information for their genetic conservation and breeding (Montilla-Bascón et al., 2013). The large number of available molecular markers and the high-density consensus map make it easier and more efficient for researchers to explore genetic diversity, population structure, and phenotype-associated QTLs in a sample of the oat germplasm.

In the present study, 2,213 high-quality polymorphic SNPs were identified among 288 oat accessions using the 6K SNP array. The reduction of genetic diversity in cereal crops, such as wheat and maize, during domestication and modern breeding has been a longstanding concern (Eyre-Walker et al., 1998; Cavanagh et al., 2013; Beissinger et al., 2016). However, in some instances, the loss of diversity was not observed from landraces to breeding accessions. Reif et al. (2005) reported that wheat genetic diversity narrowed down from 1950 to 1989 but was enhanced from 1990 to 1997. A similar increase in the average genetic diversity was detected in oat cultivars released between 1930 and 1950 (Fu et al., 2003). Oat landraces and cultivars used in this study showed similar genetic diversity, and a decreasing trend of genetic diversity was not observed from landraces to cultivars. One plausible explanation for this observation is that oat has a relatively short modern breeding history compared with other main cereal crops, and therefore has not experienced intense artificial selection (Yan et al., 2020). Another main reason for the result is that breeders used varied and geographically diverse oat resources during breeding programs. This extensive hybridization not only increased oat yields but also, simultaneously, broadened the genetic background of the cultivars (Fu et al., 2003; Yan et al., 2020). Oat accessions from different regions exhibited varied genetic diversity. Previous studies revealed that the genetic diversity of oat lines originating in Europe was lower than that of lines originating in Northern and Southern America (Fu et al., 2005; Achleitner et al., 2008); our results showed a similar trend (**Table S6**). The superior genetic diversity of oat lines from America was closely related to oat domestication, spread, and breeding history. The oat accessions of America were originally brought from Europe by humans. Later, germplasm exchange of oat took place frequently, and more exotic varieties (other species or ecotypes, even wild resources) originating from worldwide locations were used in breeding programs (Rodgers et al., 1983). The introgression of exogenous genes greatly increased the genetic diversity of American oat accessions.

Strong population structure has been reported within the germplasm of other crops. For example, Muñoz-Amatriaín et al. (2014) separated barley accessions into distinct subgroups, based on the row number (2 vs. 6), growth habit (spring vs. winter), hull type (hulled vs. hulless), improvement status (wild, landrace, and cultivar), and geographical origin. Oat also has four recognizable characteristics, including hulled, hulless, spring, and winter. Unfortunately, most previous studies reported the oat population structure as weak, and could not use any morphological trait to divide the oat accessions into distinct subpopulations (Montilla-Bascón et al., 2013; Esvelt Klos et al., 2016; Winkler et al., 2016). PCA revealed that the first three PCs accounted for 23.8% of the genetic variation in a 635-member CORE panel of elite oat germplasm (Esvelt Klos et al., 2016), and the first five PCs explained 25.8% of the variation in an 805-member global panel of oat lines (Yan et al., 2020). In these studies, the subgroups revealed by PCA or model-based K-means clustering overlapped and diffused. By contrast, in the USDA collection of 759 oat landraces and historic cultivars, PC1–3 together explained 38.8% of the marker variation, and the majority of oat lines clustered into three subgroups. The population structure pattern was strongly associated with the lemma color and geographical origin of oat lines (Winkler et al., 2016). In the present study, PC1–3 accounted for 30.33% of the genetic variation, supporting a relatively distinct population structure. The majority of 288 oat accessions were divided into two subpopulations. The distribution of the two subpopulations in the 2-D scatter plots was nonoverlapping, and the relationship among oat lines within each subpopulation was relatively tight (**Figure 3**). The strong association of population structure with geographical origin was especially prominent among landraces (**Figure 2**), echoing the findings of Fu et al. (2005) and Winkler et al. (2016). Such a distribution pattern could probably be explained by the domestication and spread of oat around the world. It is widely accepted that oat originated in the Mediterranean region, with Turkey as its center of genetic diversity (Loskutov, 2008). Oat was then brought to Europe and Asia (Newton et al., 2011). Subsequently, cultivated oat accessions were introduced into America by the Spanish and British explorers (Rodgers et al., 1983). In the present study, POP1 mainly consisted of landraces from Western Asia (Turkey) and its circumjacent regions (Southern Asia and Southern Europe), while other landraces in POP2 were from regions farther away from Western Asia (Eastern Asia, Eastern Europe, and South America), which is concerning. The other point of concern is that at K = 2, six of the fourteen hulless oat lines from China were identified as admixtures, while the other eight hulless lines clustered into POP2 together with the hulled landraces and cultivars from China, even though their values of membership probability were not high. At K = 6, all 14 hulless lines were identified as admixtures (data not shown). In our study, all the hulless oat lines were cultivars. Pedigree information suggests that most Chinese hulless cultivars have been selected from crosses between hulless and hulled accessions (Ren and Yang, 2018; Yan et al., 2020). Therefore, the population differentiation between Chinese hulless oat cultivars and Chinese hulled oat accessions was dramatically weakened, and hulless cultivars showed a high level of admixture or proximity to common oats (Yan et al., 2020).

### 4.2 GWAS for hullessness and lemma color

During genome-wide association analysis, it is necessary to determine the density and coverage of markers according to the extent of LD that affects the power and resolution of GWAS in a given population. In the current study, the LD decay results (genome-wide average LD = ~1.4 cM) suggested that at least one marker per 1.4 cM would be necessary to perform effective GWAS in the oat population, similar to previous studies (genome-wide average LD = ~1.5 cM) (Newell et al., 2011; Yan et al., 2020). Given that the total length of the oat consensus map estimated by Chaffin et al. (2016) is 2,843 cM, the number of markers required for the oat population used in this study was approximately 2,000. Therefore, we performed GWAS using 2,143 polymorphic SNPs, which surpassed the minimum number of SNPs required and were sufficient.

Cultivated oats are generally classified as hulled and hulless types, depending on their grain phenotype. The hull of a hulled oat variety is thick, lignin-rich, and hard-to-remove, whereas that of a hulless accession is papery-thin and free-threshing (Yan et al., 2020). Previous studies demonstrated that the hulless trait in oat is controlled by a single, incompletely dominant gene (*N1*) interacting with modifying genes (Boland and Lawes, 1973). The *N1* locus was mapped by De Koeyer et al. (2004) to linkage group TM_5 (Terra × Marion), which was homologous to KO_24_26_34 and was later afirmedto be located at approximately 200 cM on Mrg21 in the consensus map (Chaffin et al., 2016). Ubert et al. (2017) mapped the *N1* locus in two recombinant inbred line (RIL) populations (UFRGS 01B7114-1-3 × UFRGS 006013-1 and URS Taura × UFRGS 017004-2), and found that the SNP markers associated with the hulless trait were located on the linkage group Mrg21 near marker GMI_ES14_c19259_657 at 212 cM. The GWAS strategy was also employed to study the hulless trait of oat. Tumino et al. (2016) found a robust association between the hulless trait at the 178.3 cM position on Mrg21. Another GWAS performed by Yan et al. (2020) found that the most significant markers affecting the hulless trait were located on Mrg21 at 205.3, 212.1, and 195.7 cM. The positions of associated markers in the two GWAS were discrete. This could be due to the small number of hulless oat lines in the mapping population used by Tumino et al. (2016), which limited the power of GWAS. In the present study, six significant SNPs were found to be associated with the hulless trait of oat. Four of the six associated SNPs were located at 194.0 and 205.7 cM on Mrg21, suggesting a major QTL between these genetic map positions (**Table 3**, **Figure 6A**). These SNPs were located close to the *N1* locus detected in the two RIL populations by Ubert et al. (2017), and to the associated SNPs found by Yan et al. (2020). Recently, Peng et al. (2022) generated a high-quality reference genome assembly of hulless common oat (AACCDD genome), and performed GWAS to identify the genomic loci contributing to the hulless trait. A strong peak associated with the trait was detected at the end of chromosome 4D, and colocalized with the *N1* locus. We mapped six SNPs associated with the hulless trait in common oat (**Table 4**). Four of the six SNPs (GMI_GBS_84661, GMI_ES01_c8241_504, GMI_GBS_67251 and GMI_ES22_c7478_431) mapped to the end of chromosome 4D, and two of these four SNPs (GMI_GBS_67251 and GMI_ES22_c7478_431) were located in the candidate region of the *N1* locus (Peng et al., 2022). These results provide further evidence suggesting that the major locus *N1* controls the hulless trait in oat. The formation of hulless grain is also observed in other crops, such as barley (*Hordeum vulgare*. L) (Taketa et al., 2008). Lemma and palea are attached firmly to the grain in hulled barley, while they can be easily separated from the grain in hulless barley. Most studies concluded that the hulled grain trait is governed by a single locus (*NUD*) in barley, and hulless barley varieties carry a loss-of-function *nud* allele. However, additional loci significantly associated with hullessness have recently been identified in barley through GWAS (Milner et al., 2019; Wabila et al., 2019). In the present study, two additional markers (GMI_ES02_lrc13788_346 and GMI_ES17_c10594_472), identified on Mrg05 and Mrg09 for the first time, were also found to be significantly associated with the hulless trait. Thus, our results suggest that the hulless trait of oat is regulated not only by the *N1* locus but also by other genes, as speculated previously (De Koeyer et al., 2004).

**Table 4:** Annotated genes located near significant SNP markers in a recently released genome assembly of common oat.

Trait	Locus	Linkage group	Position (cM)	Gene ID containing the SNP	Protein ID	Description	Mapping details in common oat	
							Chr.	Nucleotide position (Mb)
Hullessness	GMI_GBS_84661	Mrg21	194	LOC100844057 (*Brachypodium distachyon*)	XP_003565107.1	cell division protein FtsY homolog, chloroplastic	4D	438.93
	GMI_ES01_c8241_504	Mrg21	194	LOC100835760 (*Brachypodium distachyo*)	XP_003565080.1	villin-2-like isoform 1	4D	435.07
							4D	388.03
	GMI_GBS_67251	Mrg21	205.7	LOC4341718 (*Oryza sativa* L. subsp. *japoinca*)	ACA09448.1	probable 4-coumarate: CoA ligase 4	4D	400.03
							4D	449.44
	GMI_ES02_lrc13788_346	Mrg5	131.6	LOC123429495 (*Hordeum vulgare* subsp. *vulgare*)	BAK05479.1	predicted protein	6A	403.72
	GMI_ES22_c7478_431	Mrg21	205.7	LOC100824236 (*Brachypodium distachyo*)	XP_003559637.1	PHD finger protein ING2	4D	449.02
							4D	307.88
	GMI_ES17_c10594_472	Mrg9	61.8	LOC100843646 (*Brachypodium distachyo*)	XP_003566307.1	calmodulin binding protein PICBP	1A	317.10
							1A	454.91
Lemma color	GMI_ES15_c2369_181	Mrg20	14.7	LOC780697 (*Triticum aestivum*)	AAY84880.1	splicing factor U2af large subunit B-like	4D	23.20
	GMI_DS_oPt-18257_376	Mrg17	53.8	–	–	–	6C	137.94
	GMI_GBS_13773	Mrg18	56	–	–	–	6C	138.18

The gene *A.satnudSFS4D01G000045*, annotated as a receptor-like kinase, was suggested to be a promising candidate for the gene controlling the hulled/hulless trait in oat (Peng et al., 2022). In accordance with the SNP annotations provided by Tinker et al. (2014), genes orthologous to those carrying the hullessness trait-associated SNPs are listed in **Table 4**. Notably, the candidate gene *A.satnudSFS4D01G000045* was not present among these ortholgous genes (**Table 4**). However, we found that the gene containing the marker GMI_GBS_67251 at the *N1* locus likely encodes 4-coumarate: CoA ligase 4 (4CL4) protein. The 4CL4 protein participates in phenylpropanoid metabolism by mediating the activation of a number of hydroxycinnamates for the biosynthesis of monolignols and other phenolic secondary metabolites (Gui et al., 2011). The rice homolog of *4CL4* (*IRAL1*/*4CL4*) is also involved in lignin biosynthesis, and mutation of *4CL4* reduces the lignin content of roots and leaves (Liu et al., 2020). Ubert et al. (2017) speculated that the reduced lignification of lemma in hulless oat maybe related to genes that regulate lignin composition and biosynthesis. Therefore, we conjecture that *4CL4* might be another candidate gene controlling the hulled/hulless trait in oat, although more evidence is needed to verify this speculation. The annotated gene of GMI_ES22_c7478_431, which was also located in the *N1* locus region, was predicted to encode the PHD finger protein ING2, which participates in the growth regulation biological process. Oat possesses a large, repeat-rich polypoid genome that has undergone extensive rearrangement. Once a better quality genome sequence of hexaploid oat becomes available, additional candidate genesmust be discovered and annotated.

The most significant SNP affecting lemma color (GMI_ES15_c2369_181) in the present study was mapped to Mrg20 (14.7 cM); this SNP was also identified by Tumino et al. (2016) and Winkler et al. (2016) using GWAS. The SNP marker was mapped to chromosome 7D in common oat (**Table 4**), and the gene containing the marker shared similarity with the gene encoding the splicing factor U2af large subunit B-like protein. Two other significant markers, GMI_DS_oPt-18257_376 and GMI_GBS_13773, were reported for the first time in this study. Both these markers were located on chromosome 6C in close proximity to each other. It is possible that this region on chromosome 6C governs lemma color in oat. Lemma color is a complex and difficult-to-interpret trait. Previous investigation of the inheritance of black and gray colored lemma in a specific hybrid population of oat revealed that black lemma color is controlled by more than two loci (Hoffman, 1999). Studies conducted to date on lemma color suggest that the expression of this trait is affected by environmental factors and epistatic effects.

GWAS in our study found several SNPs associated with hullessness and lemma color in the common oat and the results provide the basis to explore the molecular mechanism of the traits in the further research. The present stuty serves as a typical example to explore genetic basis in other quality and quantity traits using GWAS strategy in common oat.

## Data availability statement

The data presented in the study are deposited in the the Genome Sequence Archivein National in Genomics Data Center , China National Center for Bioinformation / Beijing Institute of Genomics, Chinese Academy of Sciences (GSA: PRJCA015511, https://ngdc.cncb.ac.cn/bioproject/browse/PRJCA015511) that are publicly accessible at https://ngdc.cncb.ac.cn/gsa.

## Author contributions

YS and LW conceived and designed the research. LW and JX performed the experiments and analyzed the data. HW, HB, TC, EY assisted in the experiments and edited the figures. LW wrote the manuscript. YS, BZ, and WC revised the manuscript. All authors contributed to the article and approved the submitted version.
