# Supplementary material for: Population structure analysis and genome-wide association study of a hexaploid oat landrace and cultivar collection
Source: Front Plant Sci. 2023 Mar 21;14:1131751. doi: 10.3389/fpls.2023.1131751 (PMC10070682; doi:10.3389/fpls.2023.1131751)
Supplement: Supplementary file 1 [file DataSheet_1.zip › supplementary materials/Supplementary_Material_LP.docx]

Supplementary Material

Population Structure Analysis and Genome-wide Association Study of a Hexaploid Oat Landrace and Cultivar Collection

Lei Wang^1,2,3^ Jinqing Xu^1,2,3^, Handong Wang^1,2,3^, Tongrui Chen^1,4^, En You^1,4^, Haiyan Bian^1,2^, Wenjie Chen^1,2,3,4^, Bo Zhang^1,2,3,5^, Yuhu Shen^1,2,3,5*^

*** Correspondence:** Yuhu Shen: shenyuhu@nwipb.cas.cn

# Supplementary Figures and Tables

## Supplementary Figures

**
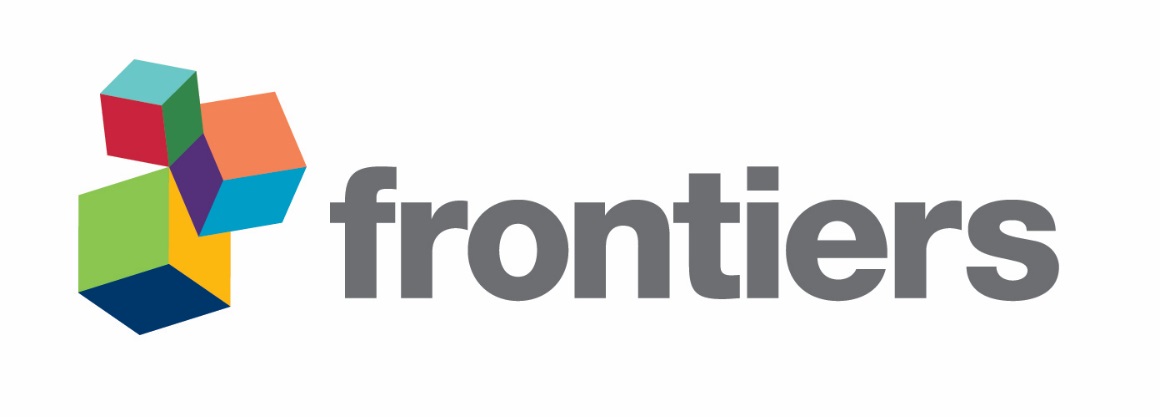
**

**Figure S1** Geographical distribution of oat accessions used in this study.


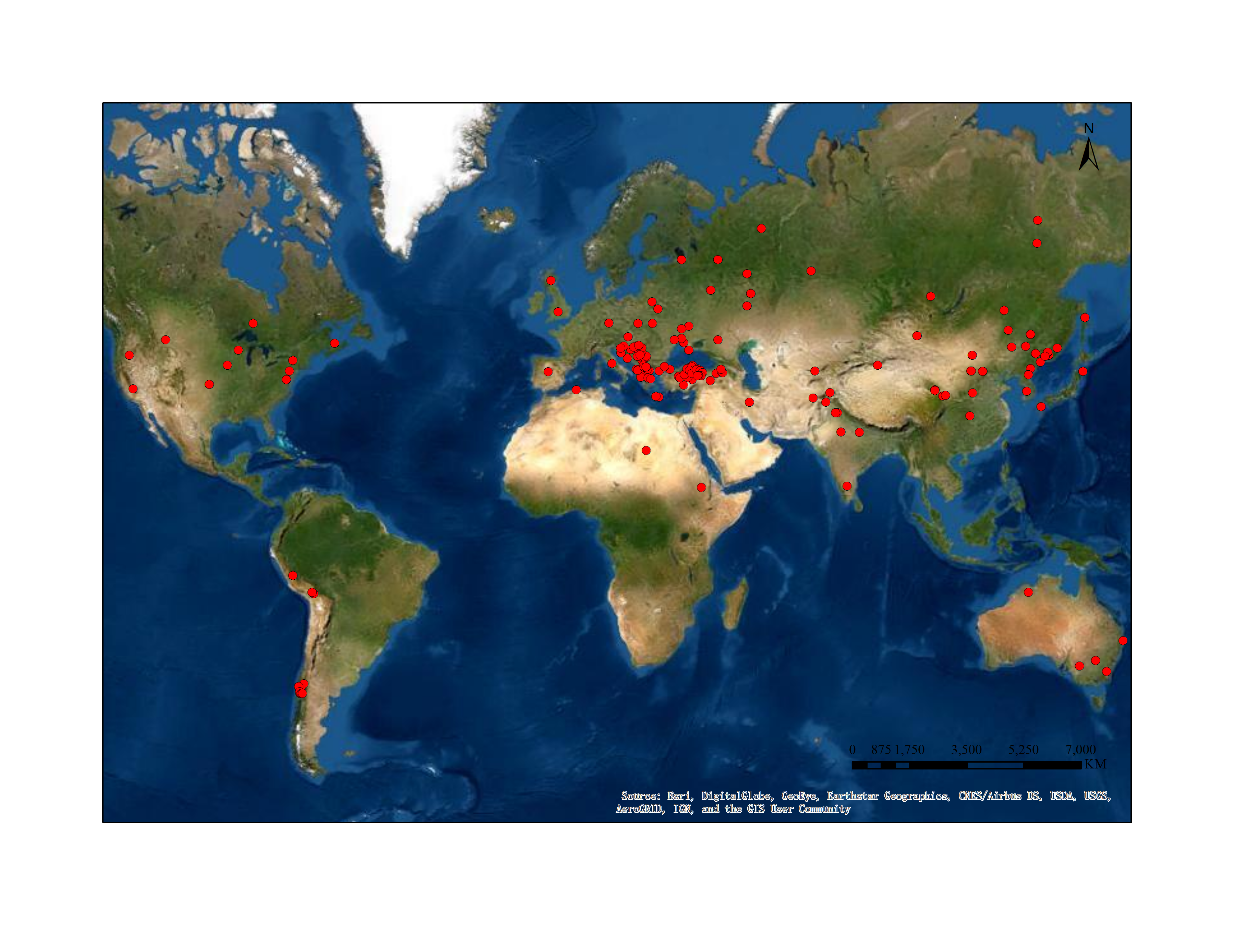


**Figure S2** Determination of the most likely value of K (the optimal population number) for 288 oat accessions. (a) Graph showing the increase in the likelihood of data, *L(K)*, at values of *K* ranging from 1 to 12. (b) Plot showing the change in ΔK used to infer the optimal K value, following the method of Evanno et al. (2005).


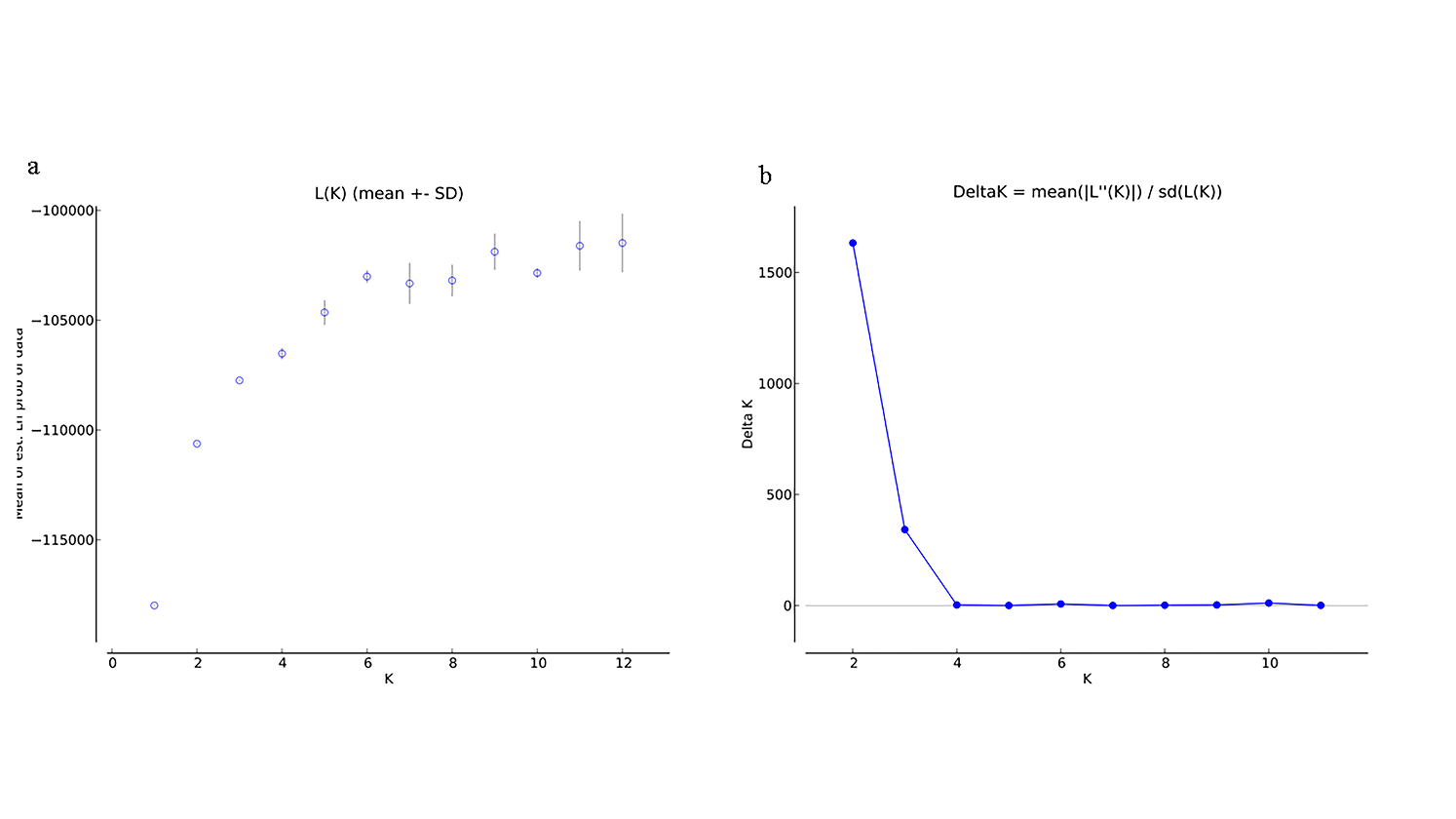


**Figure S3** Heatmaps displaying linkage disequilibrium (LD) based on r^2^ values.


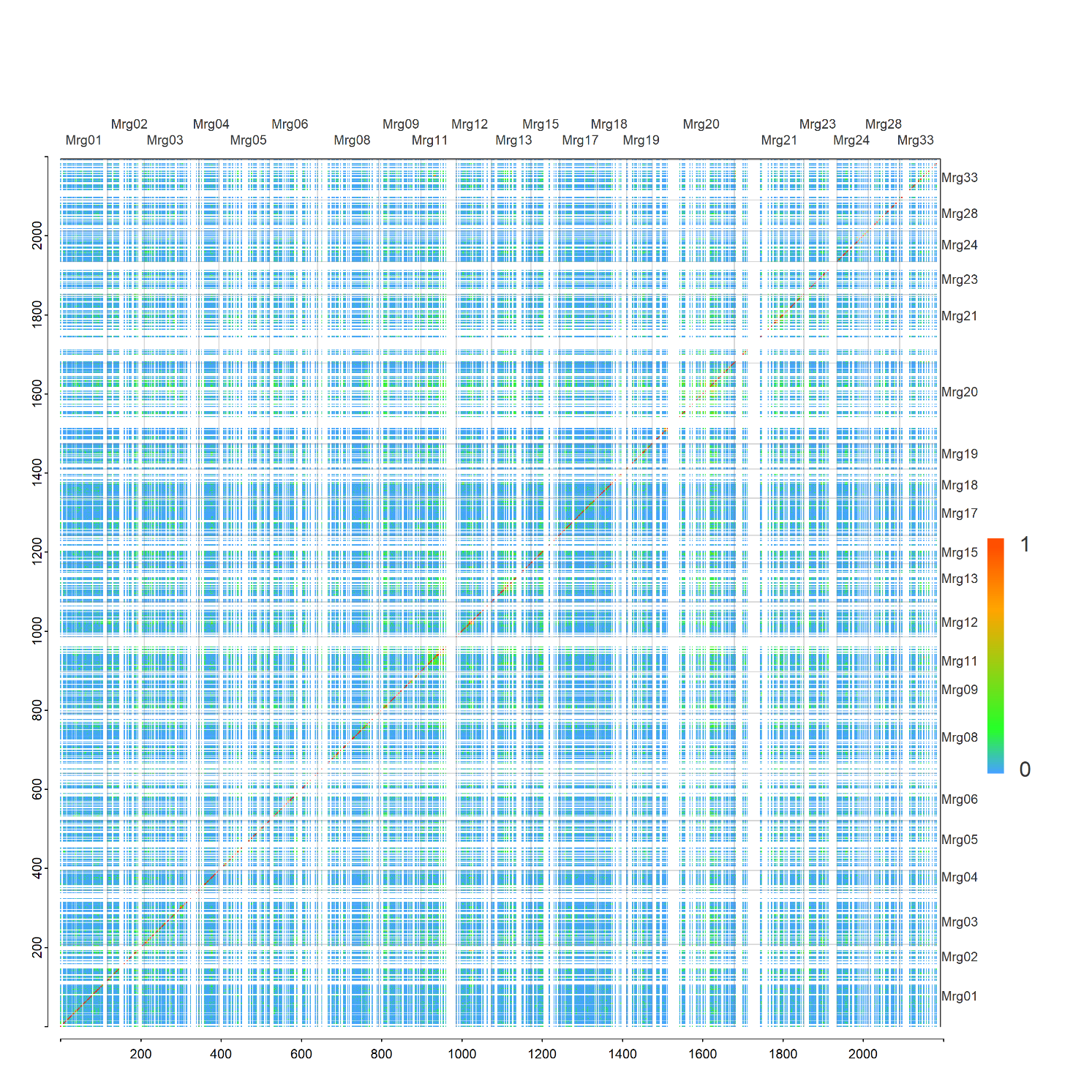


**Figure S4** Linkage disequilibrium (LD; r^2^) decay plot of SNP marker pairs with known genetic map positions (cM) in 288 oat accessions. The curve illustrates LD decay based on the nonlinear regression of r^2^ on genetic distance.


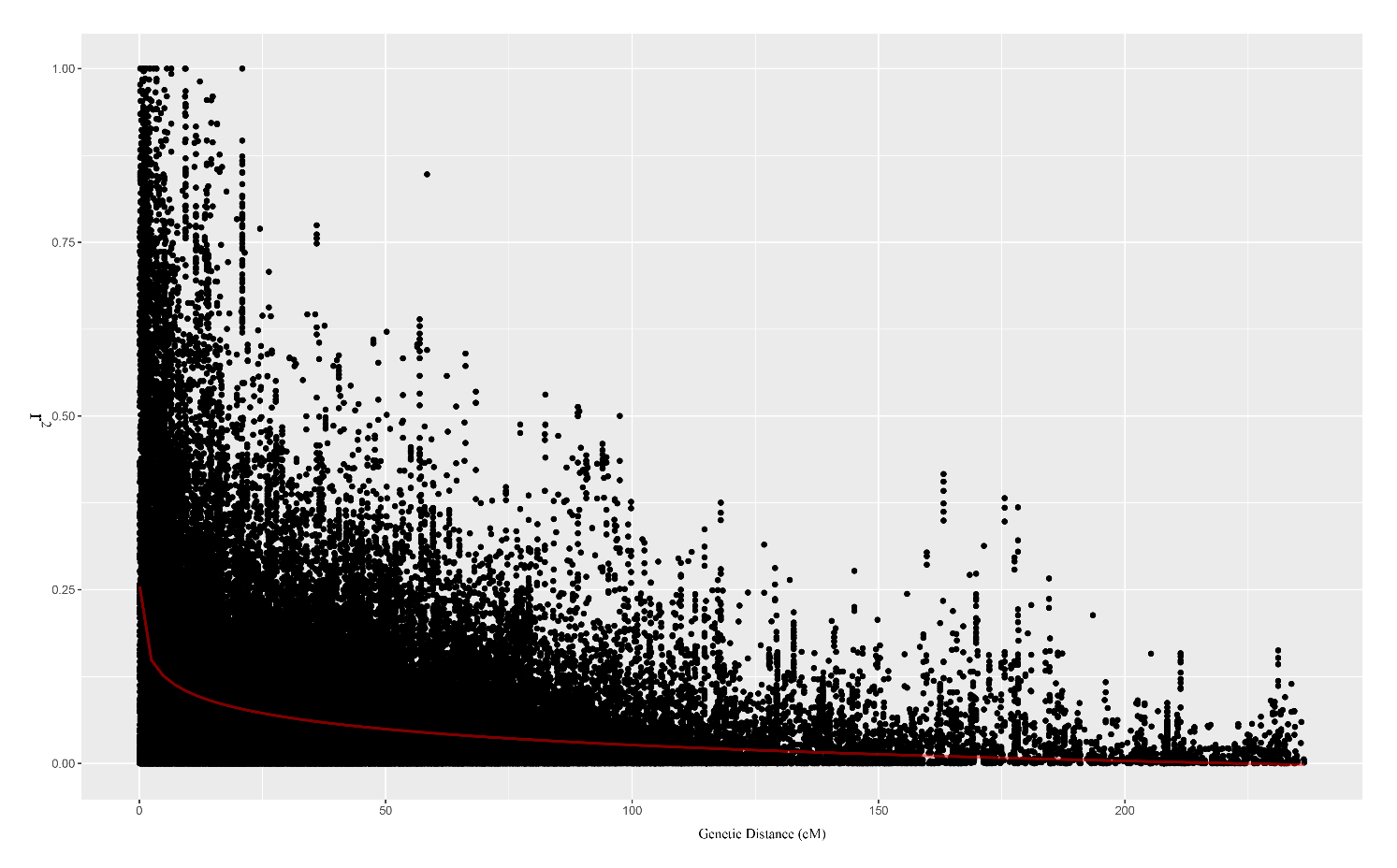


## Supplementary Tables

**Table S2.** Correlation between the population membership coefficients of oat accessions and their improvement status, year of receipt, region of origin and its coordinates (latitude and longitude), and hullessness and lemma color traits (*P* ≤ 0.01).

| Attribute of oat accessions | Population 2 (POP2) | Population 1 (POP1) |
| --- | --- | --- |
| Improvement status | -0.08431 | 0.084291 |
| Year the materials were received | -0.36652 | 0.366513 |
| Origin region | -0.49832 | 0.498312 |
| Latitude | 0.312399 | -0.3124 |
| Longitude | 0.141872 | -0.14186 |
| Hullessness | 0.036335 | -0.03634 |
| Lemma color | 0.323113 | -0.32312 |

**Table S3.** Stratification of 288 oat accessions based on the neighbor-joining (NJ) tree.

| Region of origin | Cluster 1 | | Cluster 2 | | Cluster 3 | |
| --- | --- | --- | --- | --- | --- | --- |
|  | **No. of landraces** | **No. of cultivars** | **No. of landraces** | **No. of cultivars** | **No. of landraces** | **No. of cultivars** |
| Eastern Asia |  | 1 | 24 | 33 | 2 | 6 |
| Western Asia | 41 |  | 3 |  | 2 |  |
| Southern Asia | 4 |  | 1 |  | 5 |  |
| Central Asia |  |  | 1 |  |  |  |
| Eastern Europe | 6 |  | 42 | 8 | 3 | 2 |
| Western Europe |  |  |  | 1 |  | 1 |
| Southern Europe | 33 |  | 3 |  | 10 |  |
| Northern Europe |  |  |  | 3 |  | 1 |
| Southern America |  |  | 10 |  | 4 |  |
| Northern America |  |  |  | 11 |  | 8 |
| Eastern Africa |  |  |  |  | 1 |  |
| Northern Africa |  |  |  | 1 |  |  |
| Oceania |  |  |  | 1 |  | 7 |
| Total | 84 | 1 | 84 | 58 | 27 | 25 |

**Table S4.** Mean r^2^ of genome-wide linkage disequilibrium (LD) and of each linkage group.

| **Linkage group** | **r^2^ > 0.1** | **r^2^ > 0.156** | **Genetic distance (cM)** |
| --- | --- | --- | --- |
| Genome-wide | 0.285 | 0.365 | 1.41 |
| Mrg01 | 0.231 | 0.322 | 0.78 |
| Mrg02 | 0.312 | 0.395 | 0.02 |
| Mrg03 | 0.233 | 0.303 | 1.05 |
| Mrg04 | 0.344 | 0.452 | 1.06 |
| Mrg05 | 0.253 | 0.338 | 3.16 |
| Mrg06 | 0.280 | 0.354 | 0.82 |
| Mrg08 | 0.237 | 0.341 | 0.08 |
| Mrg09 | 0.249 | 0.342 | 0.67 |
| Mrg11 | 0.316 | 0.365 | 10.11 |
| Mrg12 | 0.289 | 0.413 | 1.65 |
| Mrg13 | 0.286 | 0.353 | 6.19 |
| Mrg15 | 0.295 | 0.383 | 1.23 |
| Mrg17 | 0.228 | 0.317 | 0.78 |
| Mrg18 | 0.267 | 0.335 | 0.26 |
| Mrg19 | 0.237 | 0.343 | 1.37 |
| Mrg20 | 0.315 | 0.387 | 14.99 |
| Mrg21 | 0.259 | 0.331 | 7.16 |
| Mrg23 | 0.351 | 0.413 | 4.55 |
| Mrg24 | 0.324 | 0.442 | 1.86 |
| Mrg28 | 0.389 | 0.473 | 1.84 |
| Mrg33 | 0.263 | 0.345 | 1.19 |

**Table S5.** Phenotype of oat accessions used in the genome-wide association study (GWAS).

| Trait | Total no. of accessions (N) | Phenotype | No. of accessions |
| --- | --- | --- | --- |
| Lemma Color | 251 | Black | 22 |
|  |  | Gray | 10 |
|  |  | Red | 31 |
|  |  | Yellow | 7 |
|  |  | White | 181 |
| Hullessness | 288 | Hulled | 274 |
|  |  | Hulless | 14 |

**Table S6. Geographical distribution and polymorphic information content (PIC) of oat accessions.**

| Region of origin | No. of accessions | PIC value |
| --- | --- | --- |
| Western Asia | 46 | 0.2548 |
| Eastern Asia | 71 | 0.2499 |
| Southern Asia | 11 | 0.2737 |
| Eastern Europe | 63 | 0.2420 |
| Southern Europe | 46 | 0.2542 |
| Northern America | 19 | 0.2693 |
| Southern America | 15 | 0.2741 |
